# Experimental investigation and predictive model of entire suction range for undisturbed granite residual soil

**DOI:** 10.1038/s41598-026-43799-9

**Published:** 2026-03-11

**Authors:** Yu Zhang, Lingjie Li, Bangyan Hu

**Affiliations:** 1 Zhejiang Province Engineering Research Center for Applied Technology of Digital Highways, Zhejiang Institute of Communications, Hangzhou, 311112 China; 2 Hangzhou Xiaoshan International Airport Co., Ltd, Hangzhou, 311207 China; 3 Intelligent Transport System Research Center, Southeast University, Jiangning, Nanjing, 211189 Jiangsu China

**Keywords:** Undisturbed granite residual soil, Soil water characteristic curve, Wet-dry cycles, Predictive model, Engineering, Environmental sciences, Solid Earth sciences

## Abstract

Undisturbed granite residual soil (UGRS) in its natural state has gained attention for its broad engineering applications. Compared to remolded soils, it closely mimics natural conditions, holds unique significance in geotechnical engineering, crucial for stability analyses, environmental protection, and infrastructure development. In this study, the soil-water characteristic curve (SWCC) of UGRS were measured using indirect methods, including Pressure plate method (PPM), Filter paper method (FPM) and Vapor equilibrium method (VEM) under different wet-dry cycles. Mercury intrusion porosimetry (MIP) was conducted to obtain the pore size distribution (PSD), from which the SWCC was derived. A key novelty of this study lies in the systematic comparison and integration of experimental and pore-structure-derived SWCCs, revealing that the combination of FPM, VEM, and MIP without performing PPM can reliably cover the entire suction range while significantly reducing testing time. Test results indicate that the combination of PPM, FPM, and VEM can cover the entire range of matric suction of UGRS. However, discrepancies arise in the overlapping section where PPM records higher values than FPM. The SWCC can be derived through calculations based on the PSD of MIP. The SWCC calculated by MIP closely aligns with PPM in the low suction range of 0-100 kPa, which is the primary range of engineering concern. The combination of FPM, VEM and MIP without the PPM is the optimal method for entire suction range SWCC determination of UGRS, ensuring data accuracy while mitigating the prolonged time consumption and high data points in the low suction section of the PPM. The SWCC of UGRS can be fitted using the Fredlund&Xing model. The fitting parameters of the SWCC of UGRS can establish a strong linear correlation with the number of wet-dry cycles, enabling the establishment of a model that predicts the SWCC of UGRS across the entire suction range by incorporating pore structure evolution and cyclic effects. The results offer an efficient and accurate alternative for full-range SWCC determination and provide a basis for predicting hydraulic behavior of UGRS under cyclic environmental conditions.

## Introduction

Granite Residual Soil (GRS) is the product of in-situ rock mass weathering, which is widespread in tropical or subtropical regions of Asia and the Americas, where weathering processes are pronounced^1^. In these areas, due to cost-effectiveness considerations, GRS is widely employed as subgrade fill material in highway construction^2,3^. GRS exhibits both typical characteristics of general soil and retains certain properties of its parent rock^4^. Conventional laboratory studies often utilize remolded soil specimens for tests, which may lead to significant discrepancies in soil properties compared to actual engineering conditions^5,6^. In practical engineering, while compacted fills are remolded, many geotechnical structures, such as cut slopes, foundations on natural ground, and excavations are constructed in or on undisturbed unsaturated soils. Moreover, even subgrade fills, once placed and compacted, are often analyzed as unsaturated materials above the water Tables^7,8^. With varying seasons and rainfall amounts, the saturation status of these soils undergoes continuous changes, accompanied by a significant increase in suction variations within undisturbed granite residual soil (UGRS)^9,10^.

The Soil-Water Characteristic Curve (SWCC) is a fundamental constitutive relationship in unsaturated soil mechanics analysis, defined as the relationship between soil moisture content (or saturation) and suction. The SWCC is employed for investigating the strength, deformation, and permeability of unsaturated soils^11–14^. Thus, accurate measurement of the SWCC is imperative. Researchers have continually enhanced their understanding of the SWCC, with numerous studies indicating that, in addition to soil type and particle characteristics, the wetting-drying cycle significantly influences the soil-water characteristic curve^15,16^.

Due to the diversity of soil types, a wide range of matric suction, varying experimental objectives, and the influence of environmental conditions, there are various methods for measuring the SWCC^17,18^. Currently, common methods for SWCC measurement include tensiometers, which is the only direct suction measurement technique, electrical or thermal conductivity sensors, thermocouple psychrometry, humidity control methods, Pressure plate method (PPM), Filter paper method (FPM) and Vapor equilibrium method (VEM), and others^19,20^. Recent advancements have enabled more efficient SWCC determination; for instance, high-capacity tensiometers allow dynamic measurement of the water retention curve under controlled evaporation rates, significantly reducing testing time while maintaining accuracy^21^. However, these methods have their respective limitations. Given the air-entry value limitation of ceramic disk, the maximum suction imposed in PPM is generally 500 kPa or 1,500 kPa^22^. FPM can cover the suction range of 10-1500 kPa and the maximum suction measured by VEM can reach 368 MPa^23,24^. Therefore, the combination of different measurement techniques is usually adopted in SWCC tests. However, because the measurement of SWCC is usually time consuming, studies on SWCC measurement across the entire suction range are limited. Additionally, whether more precise and time-efficient testing protocols can be developed, particularly for UGRS under wet-dry cycles remains an open question.

Under typical field conditions, granite residual soil used as subgrade fill exhibits a low air-entry value (generally on the order of tens of kPa), and matric suction rarely exceeds 1–2 MPa^16^. Therefore, the suction range of primary engineering relevance lies below 1500 kPa. Accurate determination of the air-entry value (AEV) is critical for SWCC characterization and subsequent unsaturated property predictions. Recent studies have demonstrated that machine learning techniques can reliably estimate the true AEV from grain size distribution curves, offering a rapid alternative to conventional laboratory methods^25^. However, the SWCC is a continuous constitutive function spanning from saturation to oven-dryness. Measuring the full suction range, including the high-suction adsorption zone is essential for establishing a theoretically complete retention model, validating MIP-derived SWCC estimation in the low suction range, and quantifying the cumulative effects of wet-dry cycles on pore structure evolution. Accordingly, this study adopts a full-range measurement approach not to imply that such suctions occur in routine engineering practice, but to serve these methodological and theoretical objectives.

Thus, it is necessary to measure the entire matric suction of UGRS for SWCC and propose predictive models for different wet-dry cycles. In this study, the SWCC of UGRS was measured indirectly using the PPM, FPM and VEM under different wet-dry cycles. MIP was conducted to obtain the PSD, from which the SWCC was also derived. Based on these experimental results, an optimal method for testing the entire suction range of UGRS was recommended, and a predictive model incorporating both pore structure characteristics and wet-dry cycles was proposed using the Fredlund & Xing framework. The findings contribute not only to the experimental methodology of unsaturated soil mechanics but also to the predictive modeling of hydraulic behavior of UGRS under realistic field conditions, providing theoretical and technical support for engineering applications involving UGRS.

## Materials and methods

### Materials

**Fig. 1:**
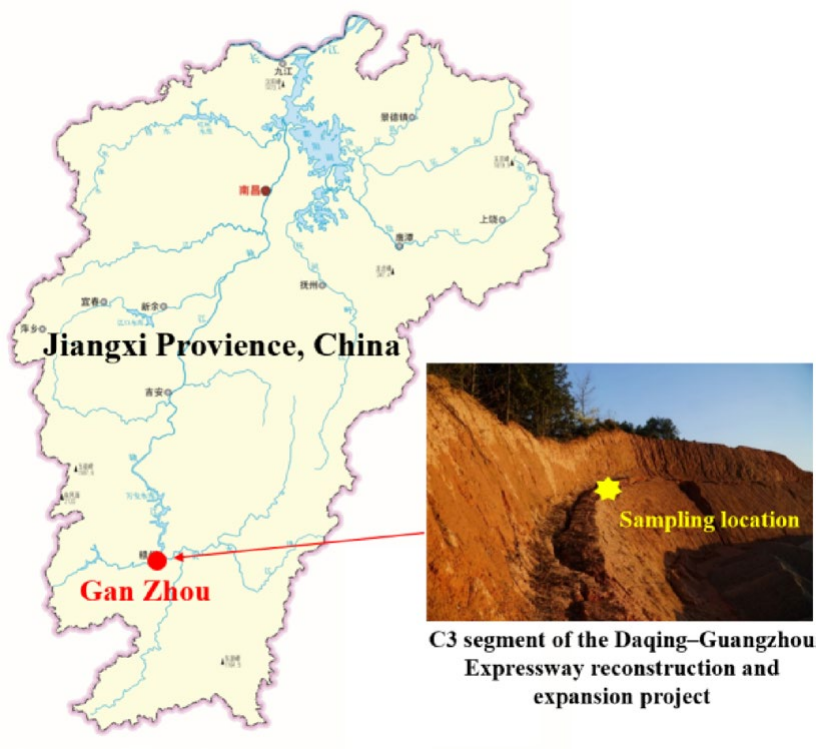
Soil sampling location.

The tested UGRS soil was sampled from the under-construction C3 segment of the Daqing–Guangzhou Expressway reconstruction and expansion project in Jiangxi Provience, China, in 2021. This segment is located in Longmu Township, Nankang District, Ganzhou City, southern Jiangxi, which lies within the typical hilly terrain of the Luoxiao Mountains range. Geomorphologically, the area is characterized as a structurally denuded low-mountain and hilly landscape, with elevations ranging from approximately 190 to 240 m above sea level and relative relief of 30–50 m. Regionally, the strata consist of Yanshanian (Mesozoic) medium to coarse grained biotite granite. Subtropical weathering has developed a 10–30 m thick profile comprising residual soil, fully weathered granite, and strongly weathered granite. Drilling revealed that the thickness of UGRS is 1–2 m thick, locally reaching 3–5 m. Soil samples were obtained taken from the first level platform of the slope, and the depth was approximately 3 m from the top of the slope, shown in Fig. 1.

The undisturbed soil samples, measuring 20 mm in height and 61.8 mm in diameter, were obtained using the “trench cutting method”. Immediately after extraction, each sample was placed into a sealed plastic bag and wrapped thoroughly with transparent adhesive tape to minimize moisture loss and disturbance. Subsequently, the samples were further encased in plastic wrap and placed into specifically designed sample containers corresponding to the ring cutter size. Each sample was clearly numbered and labeled. It is worth noting that, due to the inherent anisotropy of the structured granite residual soil, particular attention was paid to distinguishing the top and bottom surfaces of the undisturbed specimens. This distinction was clearly marked immediately after packaging to ensure proper orientation during subsequent testing.

A classification test indicated that this material, characterized by its reddish-brown color, was Clayey Sand (SC) based on the Unified Soil Classification System^26^. Compaction tests were also conducted to determine the maximum dry density and corresponding optimum moisture contents^27^. The mechanical and physical properties of the soil for experiments are summarized in Table 1.

**Table 1:** Mechanical and physical properties of the test soil.

Soil type	SC
Natural moisture content (%)	22.1
Maximum dry density (g/cm^3^)	1.79
Optimum moisture content (%)	12.1
Specific gravity	2.60
Liquid limit (%)	45.0
Plasticity index (%)	29.0
Percent of grains passing through the 4.75 mm (No.4) Sieve (%)	100
Percent of fine grains passing through the 0.075 mm (No.200) Sieve (%)	47.26

**Fig. 2:**
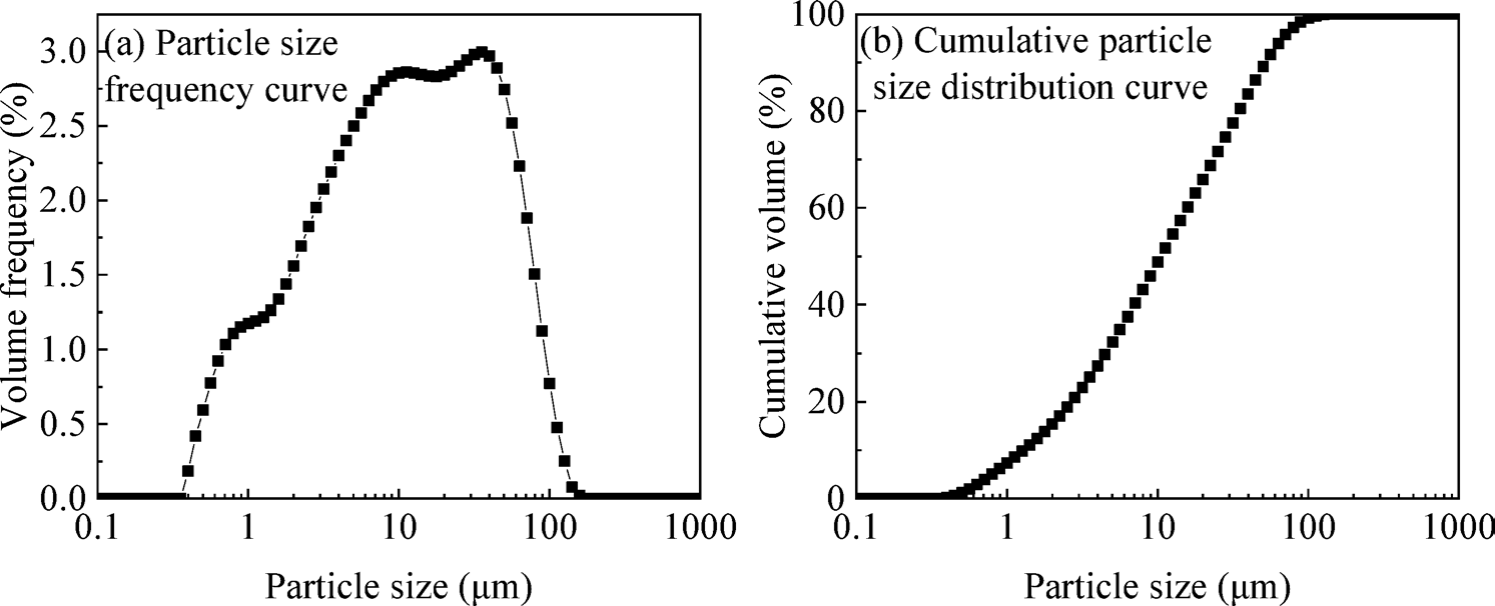
Particle size distribution curve of UGRS.

Figure 2 presents the particle size distribution curve of the UGRS used in this study, which was obtained using a laser diffraction particle size analyzer. The figure includes both the particle size frequency curve by volume and the cumulative particle size distribution curve.

### Specimen preparation

The lower bound of moisture content during drying was controlled at approximately 12%, based on the historical meteorological data of the sampling location and long-term temperature and humidity monitoring on site^28^.The upper bound corresponds to the saturated moisture content, which is not constant but progressively decreases with increasing wet-dry cycles due to cumulative pore structure changes, such as microcrack development and particle rearrangement. Accordingly, the moisture content variation in each cycle ranges from this controlled lower bound to the cycle-dependent saturated state.

**Fig. 3:**
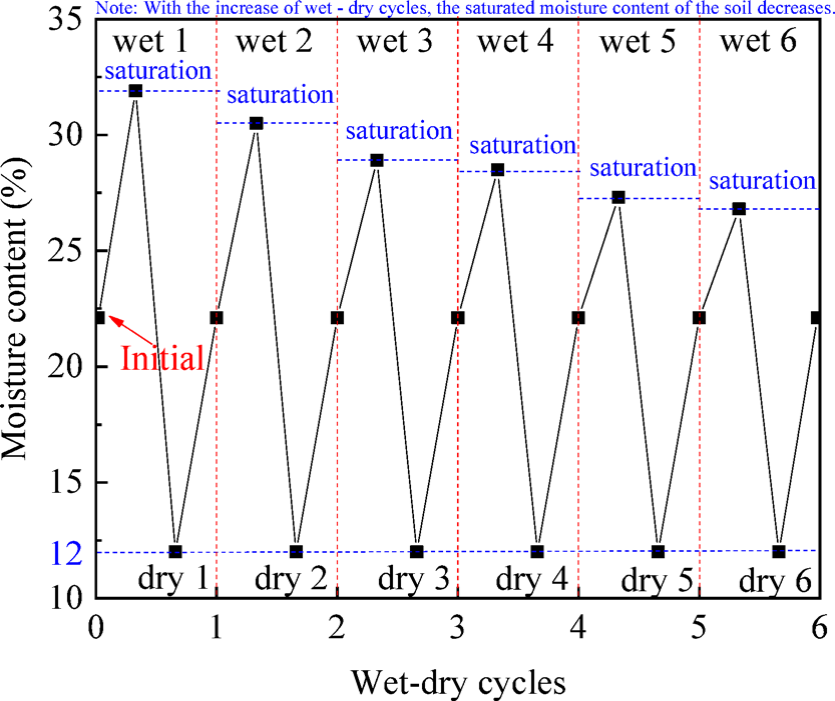
Changes in moisture content during wet-dry cycles.

Each wet-dry cycle commenced with the specimen at its natural moisture content. Vacuum saturation was first employed to saturate the samples for a minimum of 24 h. Subsequently, a dehumidification process was conducted using a constant temperature air-drying cabinet set at 45 °C. This temperature was selected based on the climatic conditions of the sampling site, where historical meteorological data indicate that extreme summer air temperatures in Nankang District, Ganzhou, can reach 37 °C. To accelerate the cyclic testing process while remaining within a thermally non‑destructive range for granite residual soil, a drying temperature of 45 °C was adopted as a reasonable compromise between field relevance and experimental efficiency. The dehumidification process was terminated once the specimen moisture content reached approximately 12%. Thereafter, a uniform water droplet application was achieved using a pipette from the specimen’s surface to restore it to the natural moisture content, thereby completing one wet-dry cycle. Previous studies have indicated that the physical and mechanical properties of UGRS, such as dry density, void ratio, and shear strength parameters, tends to reach a relatively stable after six wet-dry cycles^29,30^. Therefore, the number of wet-dry cycles applied in this study was set to six, and the specific variation in moisture content is depicted in Fig. 3.

It is acknowledged that drying at 45 °C and dropwise rewetting do not fully replicate natural evaporation and rainfall infiltration. This controlled laboratory procedure is intended as an accelerated and reproducible method to evaluate the cumulative effects of wet-dry cycles on soil pore structure and hydraulic behaviour, rather than a direct simulation of field conditions.

### Tests methods

#### Pressure plate method (PPM)

**Fig. 4:**
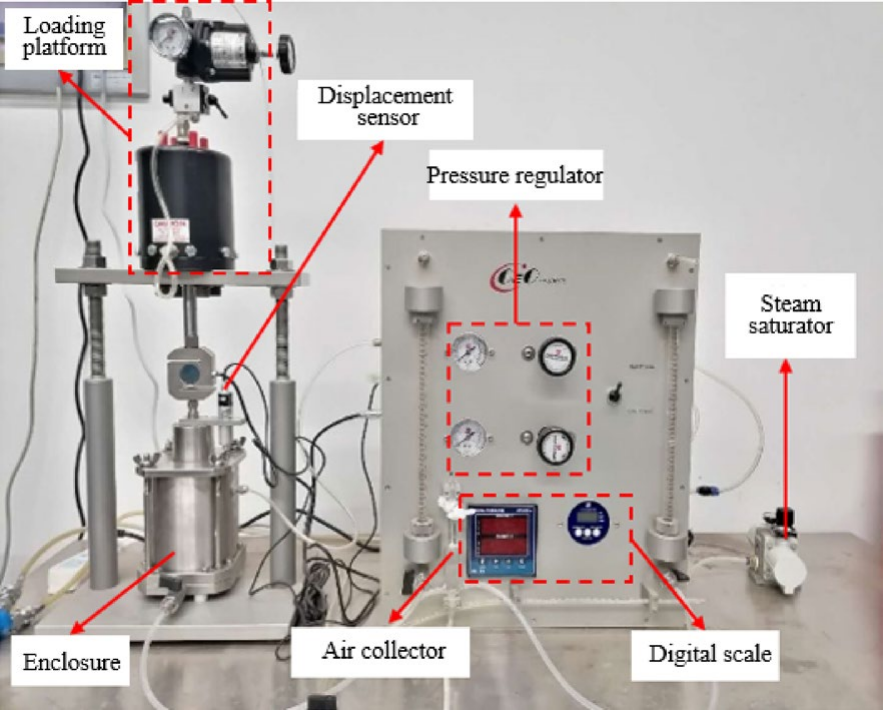
Apparatus used in pressure plate method (PPM) test.

The pressure plate method (PPM) is based on axis translation technology. The apparatus used was a GEO-Experts pressure plate system, which incorporates a loading frame, displacement sensor, pressure regulator, steam saturator, enclosure, air collector and digital scale to allow for volume change measurements during drying and wetting paths (Fig. 4). After saturation, the contraction film established between the clay plate and the air in the soil effectively hinders the passage of gases, permitting only the movement of water within the soil specimen.

Prior to testing, the ceramic disk was saturated by immersing it in de-aired water under vacuum. The soil specimen was then placed on the saturated ceramic disk to ensure hydraulic continuity. The chamber was sealed, and the desired matric suction was applied by increasing the air pressure inside the chamber via the pressure regulator, while the pore-water pressure was maintained at atmospheric pressure through the water drainage line. This pressure difference causes water to drain from the specimen, passing through the ceramic disk and into the air collector, where the outflow volume was measured using the digital scale. Throughout the process, the vertical displacement of the specimen was continuously recorded by the displacement sensor, and the axial stress was maintained by the loading frame to simulate specific overburden conditions. Once outflow ceased and equilibrium was reached, the applied suction corresponded to a point on the SWCC. This procedure was repeated for a series of incremental suction steps until the desired range was covered.

#### Filter paper method (FPM)

The FPM was conducted using Whatman No.42 filter paper (φ47 mm) and a common type of qualitative filter paper (φ55 mm) was selected as protective cover. The Whatman No.42 filter paper was sandwiched between two protective papers to prevent contamination and these three papers were sandwiched in the middle of a stack of two specimens.

**Fig. 5:**
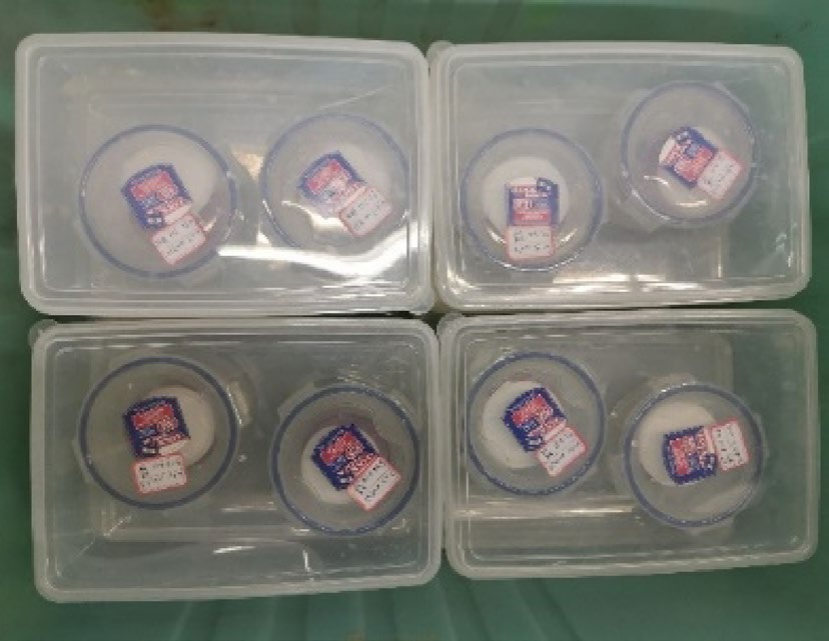
Insulated box in FPM test.

The prepared specimens were then put in a plastic container, which was sealed with plastic tape. All of the specimens were placed in an insulated box, where the temperature was controlled by air conditioners at about 20 °C for 14 days, as shown in Fig. 5. After that, the moisture content of the filter papers was measured and the matric suction of specimens could be calculated by the suction calibration Eq. (1)^31^. The specimens with natural moisture content were conditioned to moisture levels of 12.1% (the optimum moisture content), 14.6%, 17.1%, 19.6%, 22.1%, 24.6%, and 27.1% through wetting or drying. These levels were achieved through either air-drying or controlled wetting. For drying, specimens were exposed to ambient laboratory conditions and periodically weighed until the desired moisture content was reached. For wetting, a predetermined amount of de-aired water was evenly sprayed onto the soil, which was then thoroughly mixed. To ensure moisture homogenization, each specimen was immediately sealed in a plastic bag and stored in a humid container for at least 24 h prior to testing. This equilibration period allowed the moisture to distribute uniformly throughout the specimen, minimizing any potential gradients that could affect the test results. These seven different moisture content levels were utilized in FPM.

1 $$\lg {s_t}=\left\{ \begin{gathered} - 0.0779{w_{fp}}+5.327,{w_{fp}}<45.264 \hfill \\ - 0.0135{w_{fp}}+2.412,{w_{fp}} \geqslant 45.264 \hfill \\ \end{gathered} \right.$$

### Vapor equilibrium method (VEM)

The vapor equilibrium method (VEM) is commonly used for high suction ranges above 10,000 kPa^32^. The selected supersaturated salt solutions and corresponding total suctions are listed in Table 2^33^. It is noted that the suction measured in VEM is the total suction. The difference between the total suction and the matrix suction can be ignored in the high suction range^34^. For simplicity, the suction measured in this method was approximated as the matric suction^35^.

**Table 2:** Saturated salt solutions and corresponding suctions (20℃).

Saturated salt solutions	Relative humidity (%)	Total suction (MPa)
ZnSO_4_	89.96	12.60
NaCl	75.47	38.00
NaBr	59.10	71.12
MgCl_2_	33.10	149.51
KCl	85.10	218.20
LiCl	12.00	286.70

**Fig. 6:**
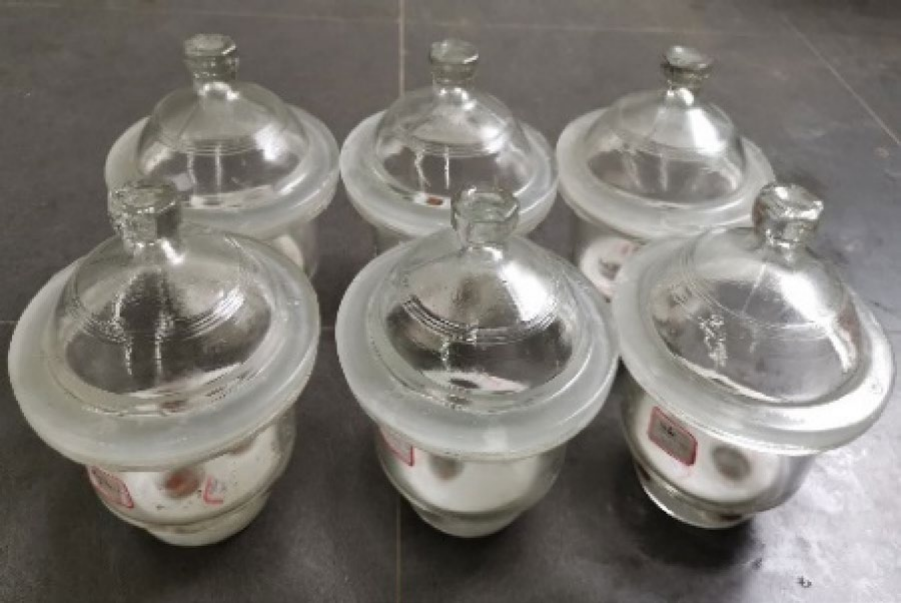
Apparatus used in VEM tests.

The specimens were placed in sealed glass desiccators equipped with a perforated plastic plate, the supersaturated salt solution was carefully poured into the bottom of the desiccators, ensuring that the solution height remained below the level of plastic plate (Fig. 6). Vaseline was also used to seal the ground glass joints.

### Mercury intrusion porosimetry (MIP) test

**Fig. 7:**
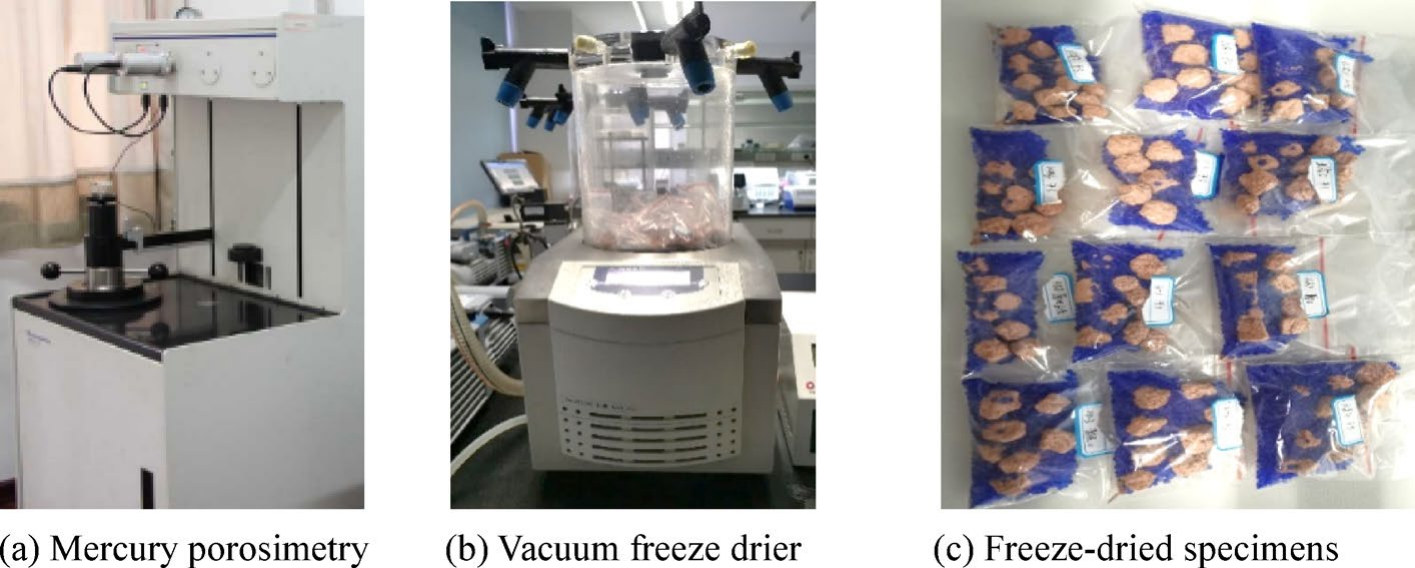
Apparatus and specimens in MIP test. (**a**) Mercury porosimetry, (**b**) Vacuum freeze drier, (**c**) Freeze-dried specimens.

The mercury injection apparatus used in this study is AutoPore IV 9510 (Fig. 7a) with a maximum intrusion pressure of 413.7 MPa and a pore size measurement range from 3 nm to 106 nm. The specimens were rapidly dried using the freeze-drying method before test. Instantaneous freezing was used to minimize the shrinkage and eliminate the surface tension generated by the curved liquid surface between the gas and liquid phases. Refrigerant was used to rapidly freeze the pore water in the specimen into solid ice crystals. The ALPHA1-2LDplus freeze-drying machine is used for freeze-drying (Fig. 7b). Firstly, the specimens were placed in an instrument containing − 196℃ liquid nitrogen, allowing the soil sample to quickly freeze at extremely low temperatures to avoid disturbance to the pore structure. The specimens were subjected to continuous vacuum pumping at -50℃ for over 24 h to facilitate thorough drying. Upon completion of the drying process, the samples were delicately extracted using tweezers, placed into sealed bags, and equipped with desiccant silica gel to prevent reabsorption of moisture by the dried specimens (Fig. 7c).

A total of 36 undisturbed soil specimens were prepared for the three testing methods employed in this study: the pressure plate method (PPM), the filter paper method (FPM), and the vapor equilibrium method (VEM). For each method, 12 specimens were allocated and further divided into four groups corresponding to 0, 1, 3, and 6 wet-dry cycles, with three specimens per group to ensure statistical reliability.

## Results and discussions

### Matric suction measurement

**Fig. 8:**
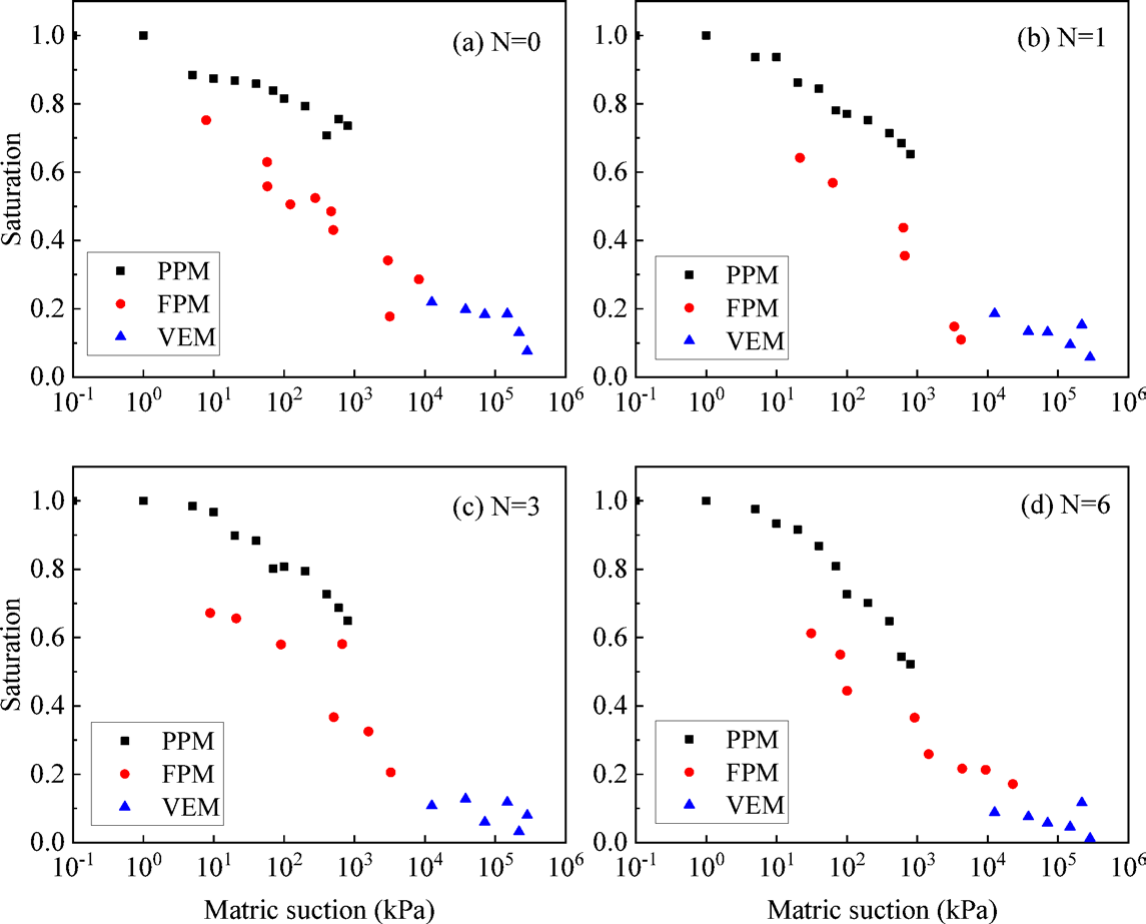
Matric suction measured by PPM, FPM and VEM of UGRS under different wet-dry cycles.

The matric measured by PPM, FPM and VEM are shown in Fig. 8. It is evident that the suction range of 0-1500 kPa can be effectively measured, demonstrating the feasibility of combining these three methods to cover the entire suction range of URGS under different wet-dry cycles. However, the test results from different methods exhibit unexpected fluctuations, particularly in the overlapping section between PPM and FPM, where the suction measured by PPM is obviously higher than that by FPM.

This discrepancy can be primarily attributed to the hysteresis of the SWCC and the distinct hydraulic paths inherent in each method^36^. Both FPM and VEM follow unidirectional paths starting from the initial state, while in PPM the specimen undergoes saturation followed by dehumidification, causing a reversal in hydraulic path that may result in significant changes in the soil pore structure^37^.

Nevertheless, it is acknowledged that factors other than hydraulic hysteresis may also contribute to the observed differences. These include, but are not limited to, variations in measurement principles (axis translation vs. dew point), calibration accuracy, sensor sensitivity, specimen preparation and handling, and the criteria used to define hydraulic equilibrium. While a systematic isolation of these equipment‑ and procedure‑related effects is beyond the scope of this study, their potential influence should be considered when interpreting the overlapping data. Future work involving inter‑laboratory comparisons or parallel tests with calibrated reference materials would help to further quantify these sources of deviation.

### Pore size distribution (PSD)

**Fig. 9:**
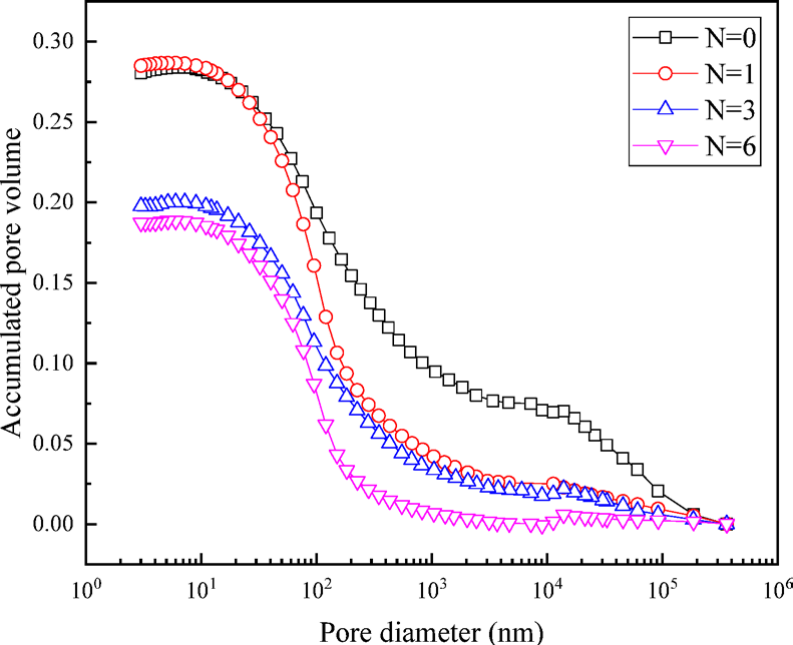
Cumulative mercury injection curve.

The cumulative mercury intrusion curves of UGRS under different wet-dry cycles are shown in Fig. 9, and the shapes of each curve are approximately similar. Notably, the mercury entry curve exhibits a significant leftward and downward shift with the increase of wet-dry cycles.

**Fig. 10:**
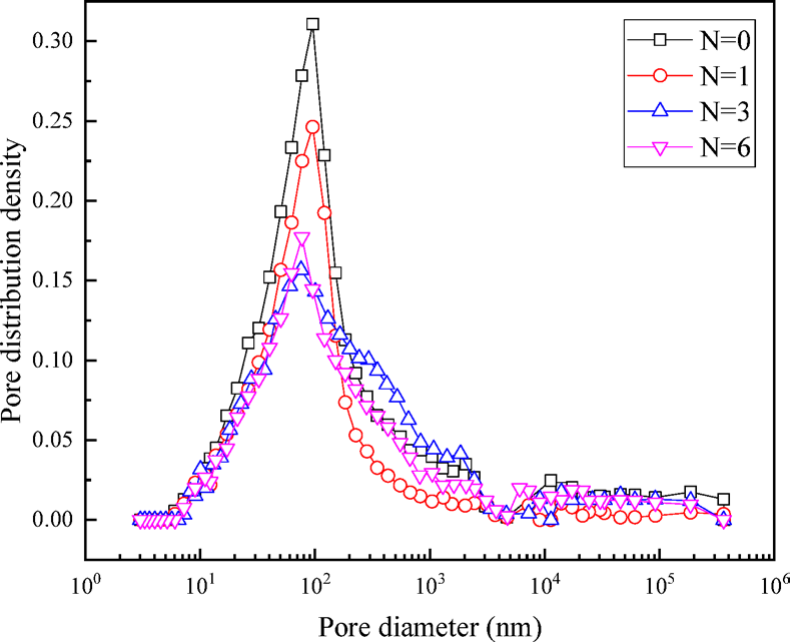
Pore size distribution (PSD).

The pore size distribution (PSD) curves of specimens under different wet-dry cycles are shown in the Fig. 10. It can be seen that the PSD presents a unimodal pore structure. The pore distribution density of specimen without wet-dry cycles is 0.31 ml/g, and the peak pore size around 95 nm. The peak pore density significantly decreases with the increase of wet-dry cycles. The number of cycles has a significant impact on the PSD, causing a shift from both sides towards the middle. In addition, the peak pore size of the specimens shifts to the right (towards the macropore direction) with the increase of wet-dry cycles, indicating the continuous development of small cracks in the soil.

### The intrinsic correlation between SWCC and PSD

The pore size measurement range of MIP is 3 nm ~ 1000 μm, which covers the suction range dominated by capillary action^38^. Scholars have attempted to link the SWCC with the PSD^39,40^. The conversion from MIP data to SWCC is based on the assumption that the pore space can be represented by a bundle of cylindrical capillaries, and that both mercury intrusion and water drainage are governed by the same capillary law. In this study, the Young Laplace equation is used to calculate the corresponding relationship between the mercury injection pressure and pore size in MIP, as shown in Eq. (2).

2 $${p_{int}}= - \frac{{4{T_{HG}}\cos {\theta _{HG}}}}{d}$$

Where *p*_*int*_ is the injection pressure of mercury, *d* is the pore size(nm), *T*_*HG*_ is the surface tension of mercury, which is 0.485 N/m at 20 ℃, *θ*_*HG*_ is the contact angle between mercury and soil interface, which refers to relevant research and takes 141.3° in this paper^41,42^.

For the moisture in unsaturated soil, the Young Laplace formula can also be used to obtain the relationship between matrix suction and pore size, as shown in Eq. 3.

3 $$s=\frac{{4{T_w}\cos {\theta _w}}}{d}$$

Where *T*_*w*_ is the surface tension of water, which is 0.0728 N/m at 20℃, *θ*_*w*_ is the contact angle between water and soil interface, which is taken as 180 ° in this paper.

Simultaneous (3) and (4), the relationship between mercury injection pressure and matrix suction can be obtained, as shown in Eq. (4).

4 $$s= - \frac{{{T_w}\cos {\theta _w}}}{{{T_{HG}}\cos {\theta _{HG}}}}{p_{int}}$$

Mercury initially infiltrates the large pores before progressing into the small ones, which is basically consistent with the process of air entering the pores of saturated soil in MIP. Due to surface tension, mercury must attain a certain pressure to enter small pores. At a given mercury injection pressure, pores larger than this critical size become filled, while those smaller remain unoccupied. Analogous to air entering saturated soil, this relationship can be expressed as follows: under a specific external pressure, pores smaller than the corresponding critical size are saturated. Based on this theory, the Eq. (5) can be used to calculate saturation in MIP. By combining Eq. (4) to convert each intrusion pressure into a corresponding matrix suction, the SWCC can be derived point by point from the MIP data.

5 $${S_{r,M}}(d)=\frac{{{V_t} - {V_d}}}{{{V_t}}} \times 100\%$$

Where *V*_*d*_ is the cumulative mercury intrusion volume corresponding to pore size d (mL), *V*_*t*_ is the total pore volume(mL), which is equal to the maximum cumulative mercury intrusion volume.

**Fig. 11:**
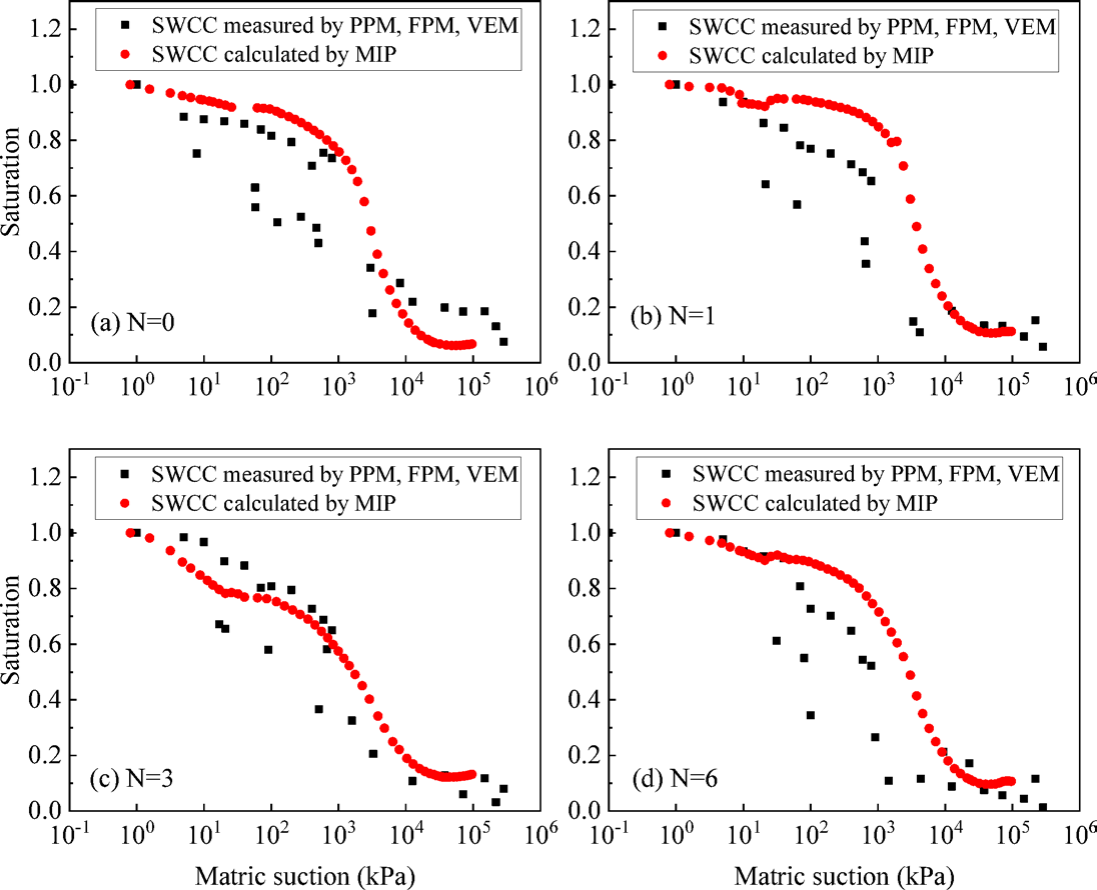
Comparison between the SWCC measured by PPM, FPM, VEM and calculated by MIP.

The comparison between the SWCC measured by PPM, FPM, VEM and the SWCC calculated by MIP of UGRS under different wet-dry cycles are shown in Fig. 11. It can be seen that within the low suction range of 0-100 kPa, the results of the two SWCCs are relatively close. As the matric suction increases, the MIP calculated SWCC is higher than the values measured by FPM in the suction range of 100-10000 kPa. After the suction exceeds 10,000 kPa, the SWCC calculated by MIP is slightly lower than the measured value by VEM.

It should be acknowledged that the conversion from MIP to SWCC involves several simplifications. First, the cylindrical capillary model idealizes the complex three-dimensional pore network; real soils contain ink-bottle shaped pores, constrictions, and tortuous pathways, which can lead to hysteresis and an overestimation of the small pore volume^43^. Second, the contact angle of water on soil particles is rarely 0° in practice, and its variability may affect the calculated suction^16^. Third, the conversion assumes that mercury intrusion and water drainage follow the same pore entry diameter, which is a reasonable first approximation but does not account for differences in wettability and fluid-solid interactions. Despite these limitations, the MIP-derived SWCC provides a rapid and valuable estimate of the water retention behavior, particularly in the high suction range where direct measurement is challenging^44^.

### Predictive model for SWCC of entire suction range for UGRS considering PSD and wet-dry cycling

The Fredlund & Xing model was adopted in this study, as previous studies have demonstrated that it exhibits the highest fitting accuracy for granite residual soils compared to other commonly used SWCC models, such as Brooks-Corey and van Genuchten^45^. Its mathematical expression is shown in Eq. (6):

6 $$\left\{ \begin{gathered} {S_r}={\mathrm{C}}\left( s \right)/{\left\{ {\ln \left[ {e+{{\left( {s/a} \right)}^n}} \right]} \right\}^m} \hfill \\ {\mathrm{C}}\left( s \right)=1 - {\mathrm{ln}}\left( {1+s/{s_R}} \right)/{\mathrm{ln}}\left( {1+{{10}^6}/{s_R}} \right) \hfill \\ \end{gathered} \right.$$

Where *s* is the matric suction (kPa), *s*_*R*_ is the residual suction (the actual maximum measurement value of 286700 kPa in this study), and a, m, n are the fitting parameters.

The Fredlund & Xing model parameters were obtained by fitting the experimental data from PPM, FPM, and VEM using a nonlinear least squares regression method. The fitting process minimized the sum of squared residuals between measured and predicted gravimetric water contents across the entire suction range. The saturated water content was determined experimentally, while the residual suction was set as the maximum measured value (286,700 kPa) from the VEM tests. The three fitting parameters were optimized simultaneously to achieve the best fit to the experimental data points.

**Fig. 12:**
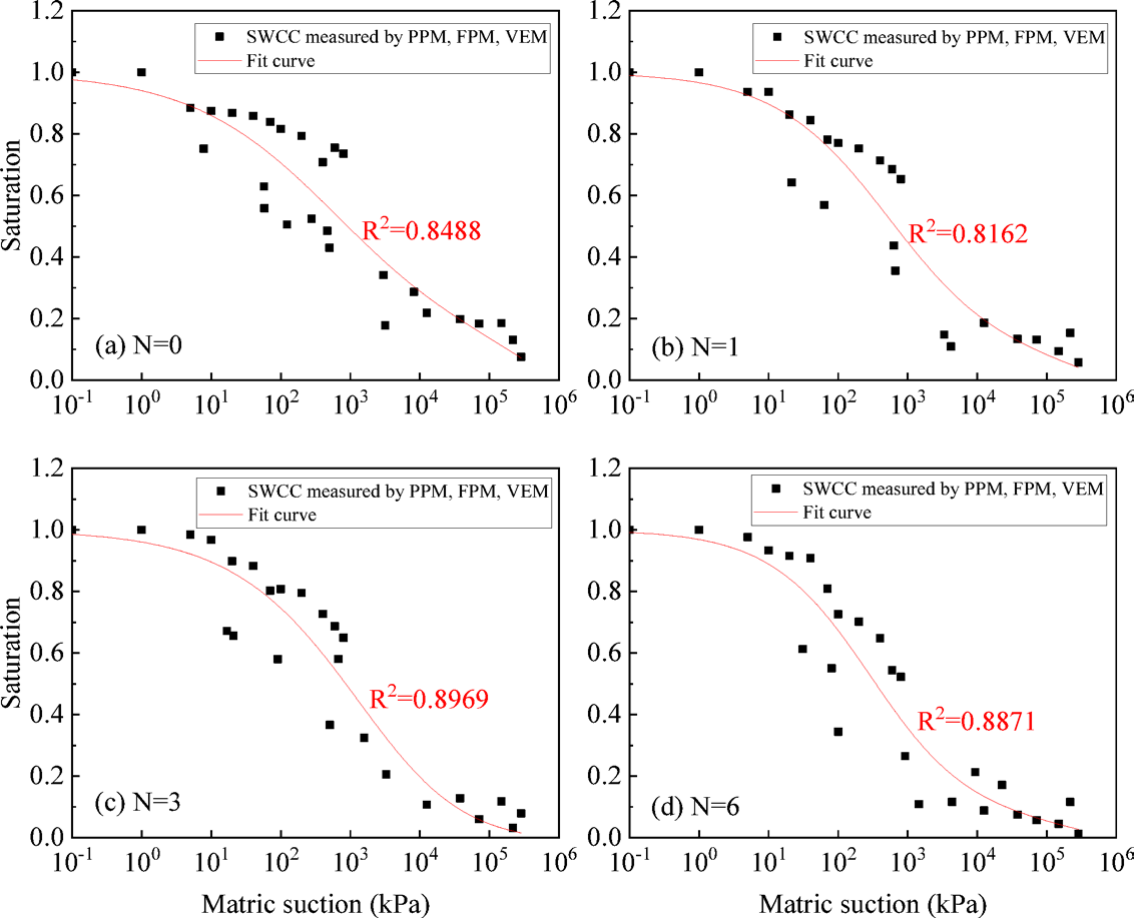
SWCC of UGRS in the full suction section by PPM, FPM and VEM.

The fitting results of the Fredlund&Xing model for the SWCC measured by PPM, FEM, and VEM are shown in Fig. 12. In the region where PPM and FPM overlap, the fitted curve consistently positions itself midway between the two, jointly determining its final configuration. The correlation coefficient R^2^ under different wet-dry cycles ranges from 0.8 to 0.9.

**Fig. 13:**
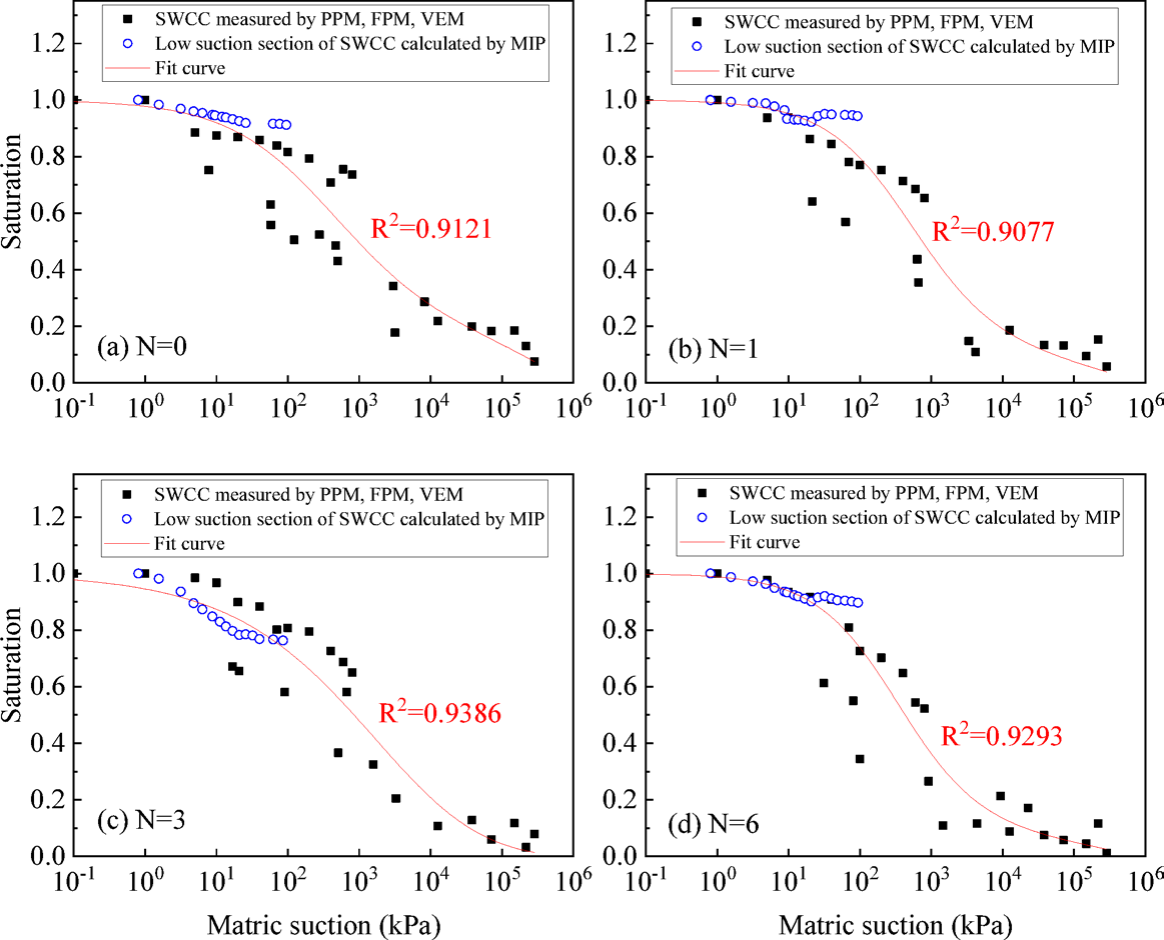
SWCC of UGRS in the full suction section by PPM, FPM, VEM and MIP.

In order to more accurately determine the SWCC, the PSD of UGRS should be considered. Therefore, the MIP reverse-calculated SWCC values within a lower suction range (0-100 kPa) were combined with PPM, FPM, and VEM data. The Fredlund & Xing model was again fitted to this combined dataset using the same nonlinear least squares optimization procedure, with the three parameters recalibrated to reflect the additional PSD information. Fitting results are shown in Fig. 13, with correlation coefficients R^2^ all above 0.90, which indicates the validity of model fitting. Therefore, considering the characteristics of soil pore structure, combing PPM, FPM, VEM and MIP can be more effective in obtaining the entire suction range of SWCC of UGRS.

**Fig. 14:**
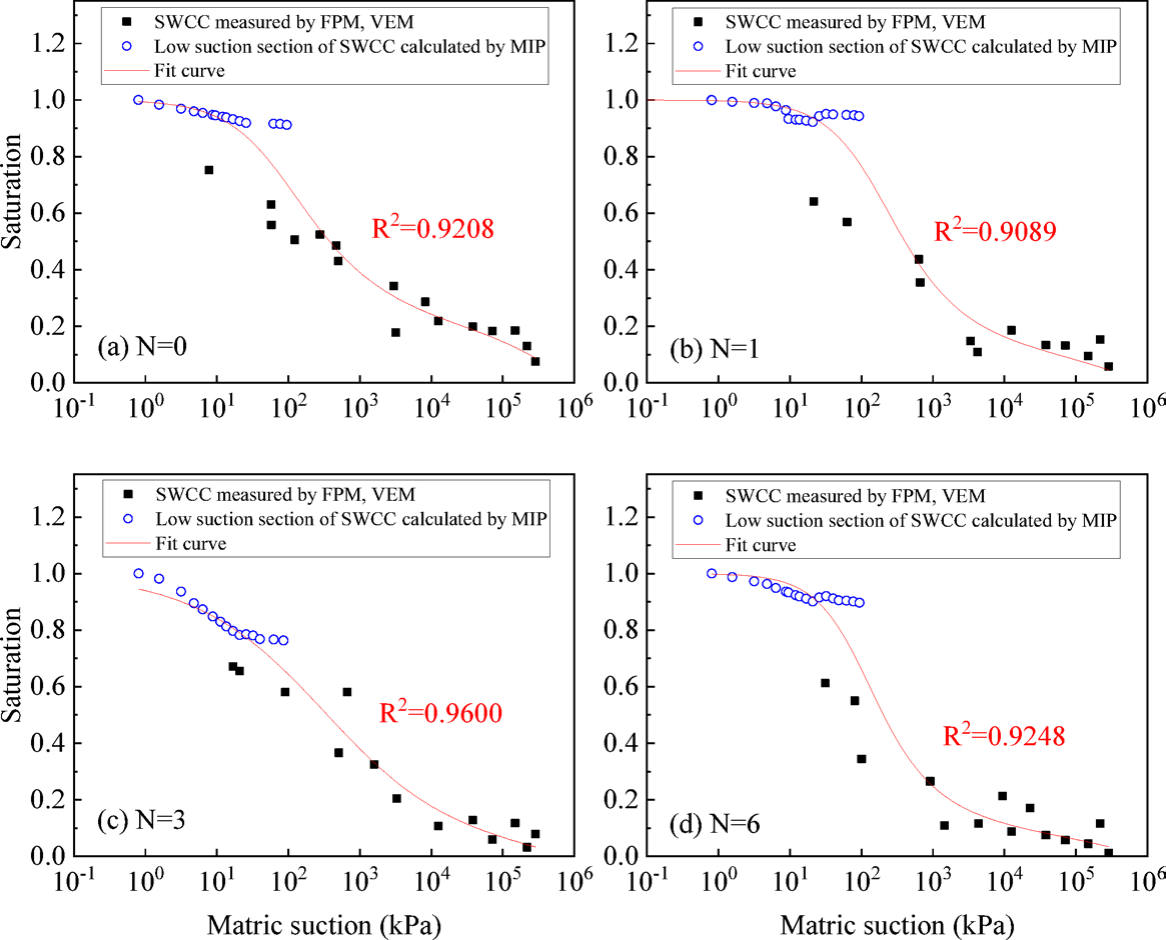
SWCC of UGRS in the full suction section by FPM, VEM and MIP.

Due to the prolonged duration required by PPM, typically around one month per specimen, it is reasonable to explore the substitution of PPM with the low suction section of SWCC calculated by MIP when time constraints arise. The Fredlund & Xing model parameters were re-fitted using only the FPM, VEM, and MIP data, following the same nonlinear regression approach. Figure 14 shows the SWCC of the full suction section measured using the FPM, VEM, and MIP without the PPM. The fitting parameters of the SWCC are listed in Table 3, the fitting accuracy closely aligns with the results obtained from all four methods. Therefore, contemplating the utilization of the low suction section derived from MIP as a substitute for PPM is a viable option. This comprehensive method can ensure data accuracy while significantly mitigating the drawbacks of prolonged time consumption and high data points in the low suction section of the PPM.

**Table 3:** Parameters of the SWCC for UGRS by FPM, VEM and MIP on Fredlund&Xing model.

Data source method	Number of wet-dry cycles	a	m	*n*	*R* ^2^
FPM WEM MIP	0	72.864	1.193	0.946	0.9208
	1	127.938	1.867	0.824	0.9089
	3	220.078	2.514	0.562	0.9600
	6	287.942	4.323	0.379	0.9248

**Fig. 15:**
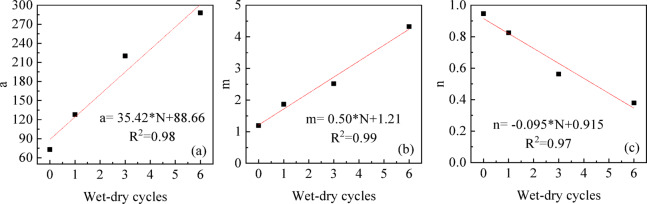
The relationship between SWCC fitting parameters and wet-dry cycles.

The relationship between the three fitting parameters of the SWCC for UGRS and the wet-dry cycles is shown in Fig. 15. As the wet-dry cycles increases, the fitting parameters a and m both increases, while the parameter n decreases.

Parameter a is related to the air-entry value of the soil. The increase in a with wet-dry cycles indicates a progressive degradation of soil structure. Cyclic wetting and drying induces micro-crack development due to repeated expansion and shrinkage of clay minerals, which weakens the soil’s water holding capacity and facilitates water drainage.

Parameter n is related to the slope of the transition section of the SWCC, reflecting the moisture reduction rate and pore-size distribution (PSD). The decrease in n with increasing cycles indicates an accelerated water loss rate, attributed to the widening of PSD as micro-cracks create a greater proportion of larger pores that drain more quickly.

Parameter m is related to the residual moisture content. The increase in m with cycles suggests a decrease in residual water content, likely due to irreversible damage to small intra-aggregate pores that retain water at high suctions. The correlation between parameter m and the wet-dry cycles exhibits the highest fitting degree, boasting an R^2^ value of 0.98. Additionally, the fitting degree of R^2^ between parameter a, parameter n and the wet-dry cycles is approximately 0.98 and 0.97. Overall, each parameter can establish a good linear correlation with wet-dry cycles.

Establish a relationship between the curve parameters and the number of wet-dry cycles, and integrate them into Eq. (6) to obtain an estimation model for the SWCC of UGRS considering wet-dry cycles, as shown in Eq. (7).

7 $${S_r}=\left[ {1 - {\mathrm{ln}}\left( {1+s/{s_R}} \right)/{\mathrm{ln}}\left( {1+{{10}^6}/{s_R}} \right)} \right]/{\left\{ {\ln \left[ {e+{{\left( {s/\left( {35.42*N+88.66} \right)} \right)}^{\left( { - 0.095*N+0.915} \right)}}} \right]} \right\}^{\left( {0.50*N+1.21} \right)}}$$

Where *s* is the matric suction (kPa), *s*_*R*_ is the residual suction (the actual maximum measurement value of 286700 kPa in this paper), and a, m, n are the fitting parameters.

**Fig. 16:**
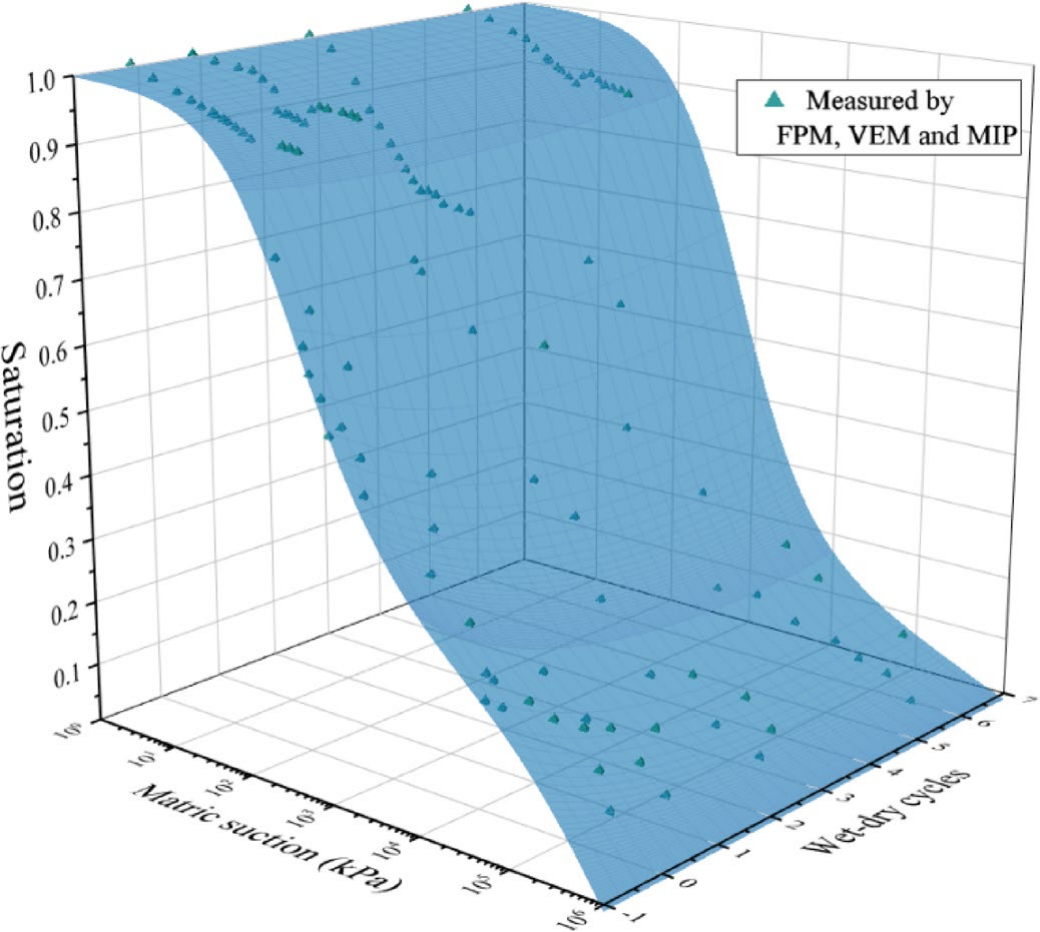
SWCC of UGRS considering wet-dry cycles.

Comparing the measured values with the estimated model in the same coordinate system, the fitting effect of the model is shown in Fig. 16. It can be seen that this model can well describe the changing trend of SWCC of UGRS under different dry-wet cycles.

## Conclusions

In this study, the SWCC of UGRS were measured using PPM, FPM and VEM under different wet-dry cycles. MIP was conducted to analyze the pore structure characteristics of UGRS, and the SWCC was also derived from MIP pore size distribution data. The SWCC obtained from MIP was compared with those measured by PPM, FPM, and VEM. The combination of FPM, VEM and MIP without the PPM is the optimal method for testing the entire suction range SWCC. Furthermore, a predictive model for fitting the SWCC of UGRS, considering pore structure characteristics and wet-dry cycles, was proposed using the Fredlund & Xing framework. The following conclusions can be drawn based on the test results and analysis:

The combination of PPM, FPM, and VEM can cover the entire range of matric suction of UGRS. However, discrepancies arise in the overlapping section where PPM records higher values than FPM.

The PSD presents a unimodal pore structure of UGRS, and the wet-dry cycles has a significant impact on the PSD. With the increase of wet-dry cycles, the continuous development of small cracks in the soil.

The SWCC derived from MIP PSD closely aligns with PPM measurements in the low suction range of 0-100 kPa, the range most relevant to engineering practice. The SWCC of UGRS can be fitted using the Fredlund&Xing model. After adding the low suction section of MIP reverse-calculated SWCC, the correlation coefficients R^2^ have increased from 0.81 to 0.90.

The combination of FPM, VEM and MIP without the PPM ensures data accuracy while significantly mitigating the drawbacks of prolonged time consumption and high data points in the low suction section of the PPM. This method is recommended for full-range SWCC determination in research contexts, whereas the MIP derived SWCC in the 0-100 kPa range offers a practical, time-efficient alternative for engineering applications.

The fitting parameters of the Fredlund & Xing model exhibit strong linear correlations with the number of wet-dry cycles, enabling the establishment of a predictive SWCC model for UGRS across the entire suction range that accounts for both pore size distribution and cyclic effects. This model allows engineers to estimate the evolution of hydraulic properties over the service life of infrastructure subjected to seasonal moisture fluctuations, without conducting extensive cyclic testing. Such predictive capability supports more realistic long-term performance assessments and informed decision-making in geotechnical design. Further study is needed to refine matric suction correction based on microstructural evolution.

## Data Availability

The datasets used and/or analyzed during the current study available from the corresponding author on reasonable request.
